# Incidental cardiac uptake in bone scintigraphy: increased importance and association with cardiac amyloidosis

**DOI:** 10.1259/bjrcr.20200161

**Published:** 2021-01-19

**Authors:** Francis T Delaney, Philip Dempsey, Ivan Welaratne, Bryan Buckley, Donagh O'Sullivan, Martin O'Connell

**Affiliations:** 1 Department of Radiology, Mater Misericordiae University Hospital, Dublin, Ireland

## Abstract

Extraosseous radiotracer uptake during bone scintigraphy must be carefully assessed and it offers the potential to detect previously undiagnosed disease processes. A range of neoplastic, metabolic, traumatic, ischaemic and inflammatory disorders can cause soft tissue accumulation of bone avid radiopharmaceuticals. Accordingly, cardiac uptake in bone scintigraphy has a broad differential diagnosis and is commonly attributed to ischaemia/infarction related to coronary artery disease. However, there has been renewed focus on incidental cardiac uptake in recent years in light of significant developments in the diagnosis and management of cardiac amyloidosis.

Cardiac amyloidosis is a form of restrictive cardiomyopathy caused by the accumulation of misfolded proteins, amyloid fibrils, within the myocardium. There are two primary protein precursors in amyloidosis, amyloid immunoglobulin light chain (AL) which causes systemic AL amyloidosis and transthyretin (TTR) which causes amyloid transthyretin (ATTR) amyloidosis. Cardiac uptake in bone scintigraphy is highly sensitive and specific for the diagnosis of ATTR amyloidosis in the correct clinical setting where AL amyloidosis has been excluded biochemically. Dedicated cardiac scintigraphy with bone avid radiotracers now plays a key role in the non-invasive diagnostic algorithm for ATTR amyloidosis and may help in its early detection.

Concurrently, multiple promising targeted therapies have emerged for ATTR amyloidosis, which previously carried a significant burden of morbidity and mortality with treatment options extremely limited. The availability of potentially disease modifying treatments has placed great emphasis on the early identification of ATTR amyloidosis. Incidental cardiac uptake during bone scintigraphy must, therefore, be accurately reported and carefully interpreted in conjunction with patient history and clinical findings as there is the potential to detect ATTR myocardial infiltration before the disease manifests clinically, as demonstrated in the cases presented here.

## Introduction

Whole body bone scan is the most frequently performed investigation in most nuclear medicine departments worldwide and second only to myocardial perfusion in the USA.^1^ Unlike many nuclear medicine tests, bone scans are not infrequently reported by general radiologists without specific fellowship training in nuclear imaging. In bone scintigraphy, radiotracer accumulates at sites of increased blood flow and bone turnover and can help to diagnose neoplastic, infectious, traumatic and other bone disorders associated with local inflammation and increased bone formation.^2^ Multiple phosphate analogues may be labelled with ^99m^Tc (99mTc) for use as radiopharmaceuticals in bone scintigraphy including 3,3-diphosphono-1,2-propanodicarboxylic acid (DPD), methylene diphosphonate (MDP), pyrophosphate (PYP) and hydroxymethylene diphosphonate (HDMP). The radiopharmaceutical is given intravenously (IV) and the average activity administered in adults is 500 Megabecquerel (300–740 MBq, 8–20 mCi). Images may be acquired in three phases – a vascular phase during IV injection, an early phase (acquired between 1 and 10 min after injection with an acquisition time of 3–5 min) and a late phase (acquired between 2 and 5 h after injection with an acquisition time of 4–10 min).^2^ Whole body images are obtained using either multiple overlapping images or continuous imaging in both anterior and posterior projections.^2^ Bone scintigraphy radiopharmaceuticals have high skeletal uptake of approximately 50% with the remainder normally cleared rapidly from soft tissues and renally excreted.^2^

Unexpected extraosseous radiotracer accumulation has been reported since bone scans were first introduced in the 1970s.^3^ Extraosseous uptake must be carefully assessed and it may occur due to a wide variety of factors such as benign or malignant disease processes, artefactual causes (such as other recent nuclear medicine investigations) or technical errors in the preparation or administration of the radiopharmaceutical – although these are rare with modern quality control measures.^4,5^ As extraosseous uptake is relatively rare and planar imaging is less familiar to current radiologists than in previous eras, an understanding of the patterns of extraosseous uptake on planar imaging and the associated causes is important for daily practice.^5^

Proposed underlying pathophysiological mechanisms for abnormal soft tissue uptake include increased local vascularity, extracellular fluid expansion and altered calcium metabolism and a range of neoplastic, ischaemic, infectious, traumatic, hormonal or inflammatory conditions may cause extraosseous radiotracer accumulation.^5–7^ Examples include certain malignancies, such as breast cancer or lymphoma, and metabolic disorders with altered calcium metabolism, such as hyperparathyroidism. Ischaemic or infarcted tissue may take up bone avid radiotracers, likely as a result of an increase in intracellular calcium, and radiotracer accumulation has been demonstrated at sites of infarction in the brain, heart and liver.^4,6^ Irreversibly damaged cells at the infarct site require some residual blood flow to demonstrate increased uptake and often after 7–10 days sites of acute infarction no longer show radiotracer uptake.^6,8^

Cardiac uptake in bone scintigraphy has a broad differential diagnosis, which includes myocardial ischaemia (myocardial infarction or unstable angina), metabolic disorders with hypercalcaemia, infiltrative diseases such as amyloidosis or sarcoidosis, inflammatory myocarditis or pericarditis, malignancy (*e.g.,* pericardial metastasis from breast cancer) and prior sestamibi imaging.^3,5,8^ Traditionally, cardiac uptake has most commonly been attributed to coronary artery disease and myocardial ischaemia with bone scintigraphy even used in the investigation of acute myocardial infarction in the past.^9^ Relatively high rates of incidental cardiac uptake have been reported in elderly patients without cardiac symptoms or a known underlying cardiac disorder for many years and the reason for this unexplained cardiac radiotracer accumulation has long been a topic of interest in the literature.^10,11^ Various aetiologies for this “benign” uptake, such as an association with prostate cancer, were proposed but its clinical significance had remained largely unclear.^7,8,10,11^

However, there has been renewed focus on cardiac uptake in recent years due to its association with cardiac amyloidosis. Cardiac amyloidosis is a form of restrictive cardiomyopathy caused by the accumulation of misfolded proteins, amyloid fibrils, within the myocardium.^12^ There are two primary protein precursors in amyloidosis, amyloid immunoglobulin light chain (AL) which causes systemic AL amyloidosis and transthyretin (TTR) which causes amyloid transthyretin (ATTR) amyloidosis. Cardiac involvement is the dominant clinical feature in ATTR amyloidosis and is also common in systemic AL amyloidosis.

Cardiac uptake in bone scintigraphy has been demonstrated to reliably detect ATTR myocardial infiltration and dedicated cardiac scintigraphy with bone avid radiotracers now plays a key role in the non-invasive diagnostic algorithm for ATTR amyloidosis and may help in its early detection.^12,13^ Uptake is typically assessed using a 4-point visual grading scale (the Perugini grade) ranging from 0 to 3 based on myocardial uptake relative to the ribs.^14^ Grade 2 or 3 myocardial uptake (uptake equal to/greater than bone uptake) is highly sensitive and specific for the diagnosis of ATTR amyloidosis in the correct clinical setting where AL amyloidosis has been excluded by demonstrating the absence of monoclonal gammopathy on serum/urine analysis.^12,13^ Accurate recognition and reporting of incidental cardiac uptake during bone scintigraphy has, therefore, become increasingly relevant and may potentially allow identification of subclinical ATTR amyloidosis before severe functional impairment occurs.

## Case report

**Figure 1.**
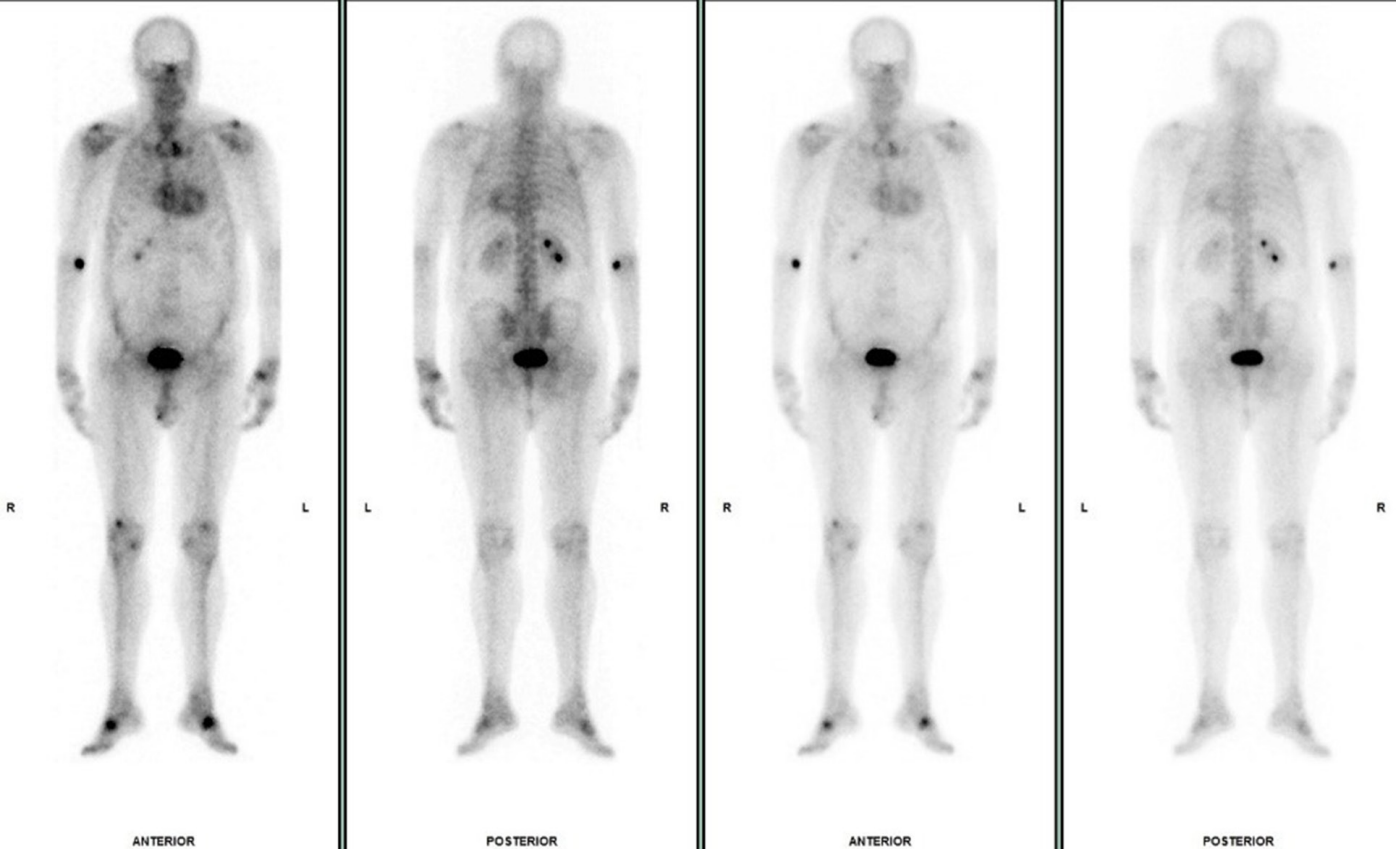
Whole body bone scan in case 1. Whole body planar images were acquired 5 min (left two images) and 3 h (right two images) after intravenous 99mTc-DPD radiotracer administration. Incidental significant cardiac uptake is seen, raising concern for cardiac ATTR amyloidosis. Otherwise, there is expected radiotracer accumulation in the skeleton and urinary tract and additional sites of focal uptake related to degenerative disease at multiple joints. Focal uptake at the injection site in the right arm is also demonstrated.

**Figure 2.**
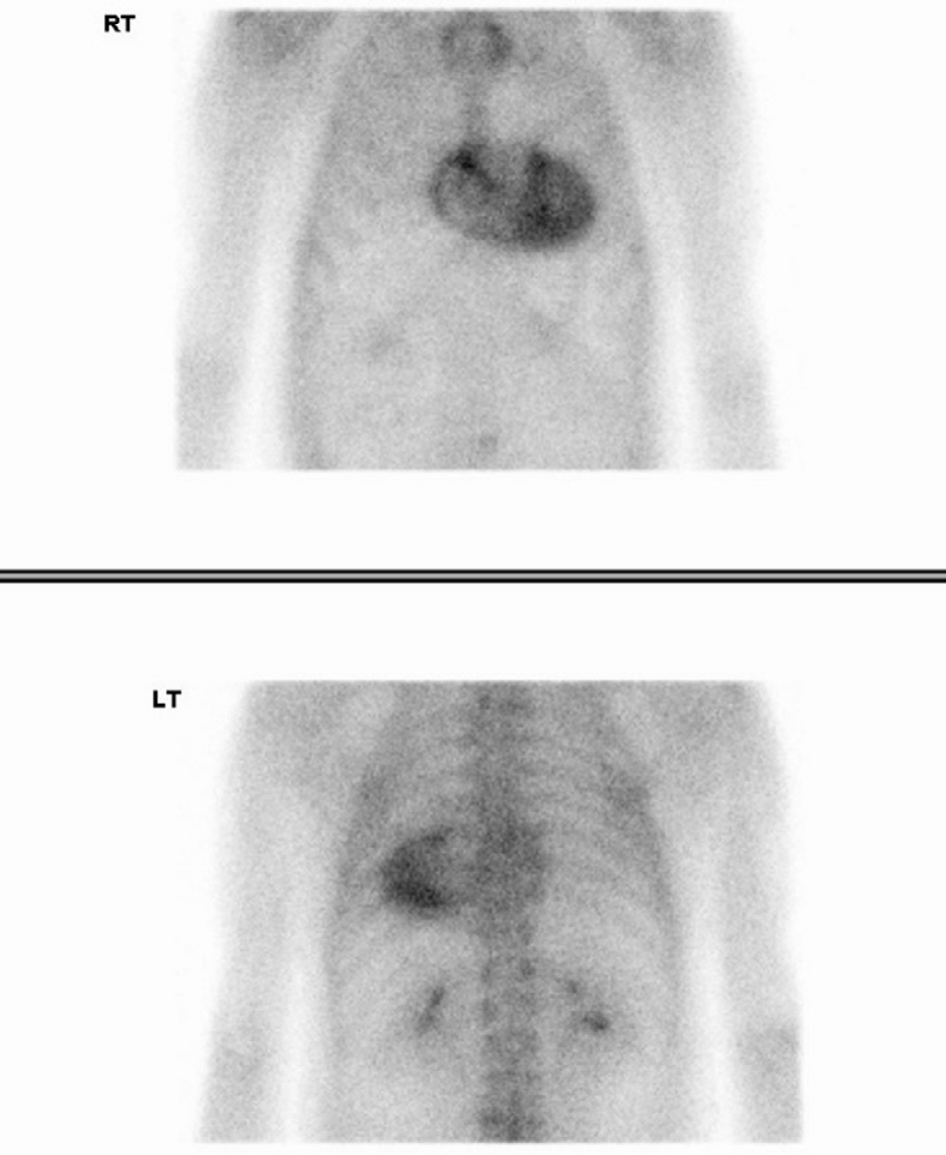
Cardiac scintigraphy in case 1. Anterior (upper image) and posterior (lower image) planar thoracic images were acquired 3 h after 99mTc-DPD administration. Severe myocardial uptake (Perugini 3 – uptake greater than rib uptake), predominantly in the left ventricle, is demonstrated compatible with the diagnosis of cardiac ATTR amyloidosis in a patient with diastolic dysfunction and absence of AL amyloidosis monoclonal gammopathy.

Case 1 is a 66-year-old male who underwent whole body bone scintigraphy with 99mTc-DPD for further investigation of a region of sclerosis in the tibia on plain radiographs. Bone scan demonstrated no significant focal radiotracer uptake related to the tibia. Diffuse cardiac uptake was identified, however, and this was included in the bone scan report and the potential association with cardiac amyloidosis suggested (Figure 1). There was no known underlying cardiac disorder at this time. Following clinical assessment, which revealed heart failure symptoms (NYHA Class 2), the patient proceeded to further evaluation with echocardiography. This showed diastolic dysfunction with preserved ejection fraction and a longitudinal strain pattern with apical sparing considered typical of cardiac amyloidosis.^12^ Dedicated cardiac scintigraphy with 99mTc-DPD was then performed, in conjunction with serum/urine analysis for monoclonal gammopathy, to distinguish between ATTR and AL amyloidosis. Cardiac DPD scintigraphy demonstrated severe cardiac uptake (Perugini 3) confirming the diagnosis of cardiac TTR amyloidosis as no evidence of monoclonal gammopathy was identified (Figure 2). The patient was commenced on novel TTR gene therapy.

**Figure 3.**
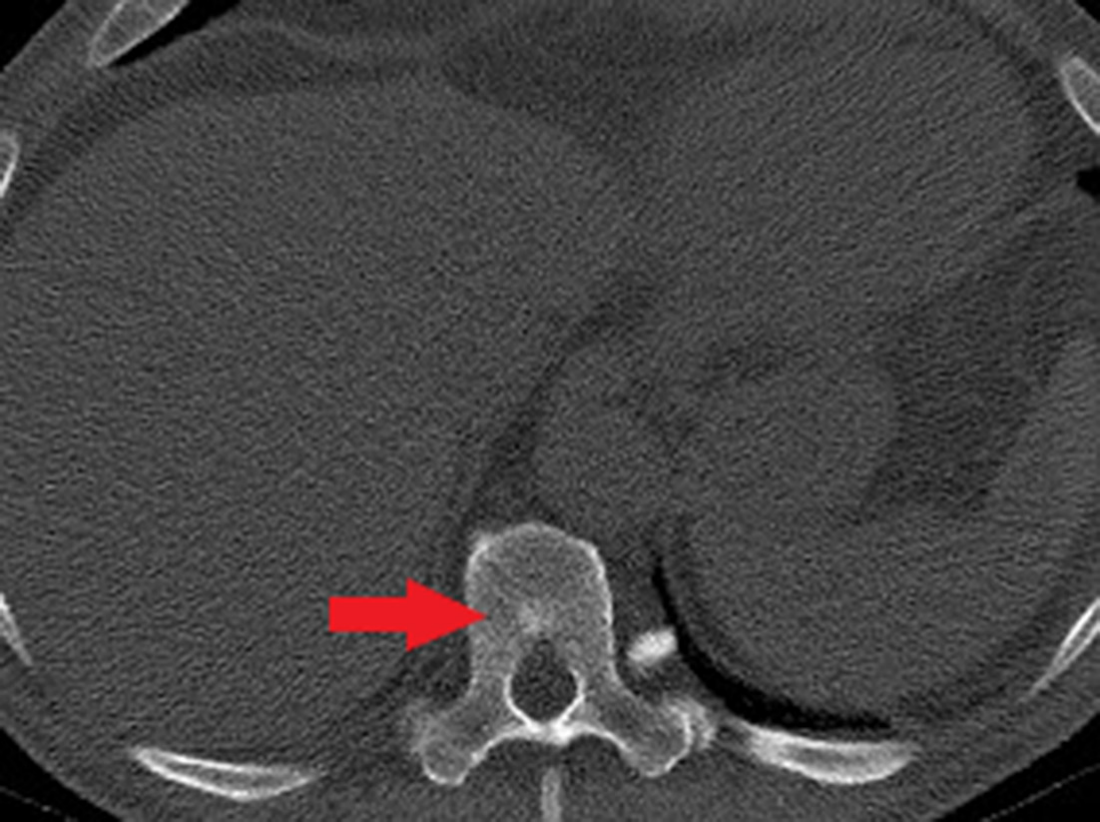
Axial CT image in case 2. Ill-defined sclerotic lesion with the T10 vertebral body (red arrow) incidentally detected on abdominal CT for an unrelated indication. Further investigation with bone scintigraphy was arranged.

**Figure 4.**
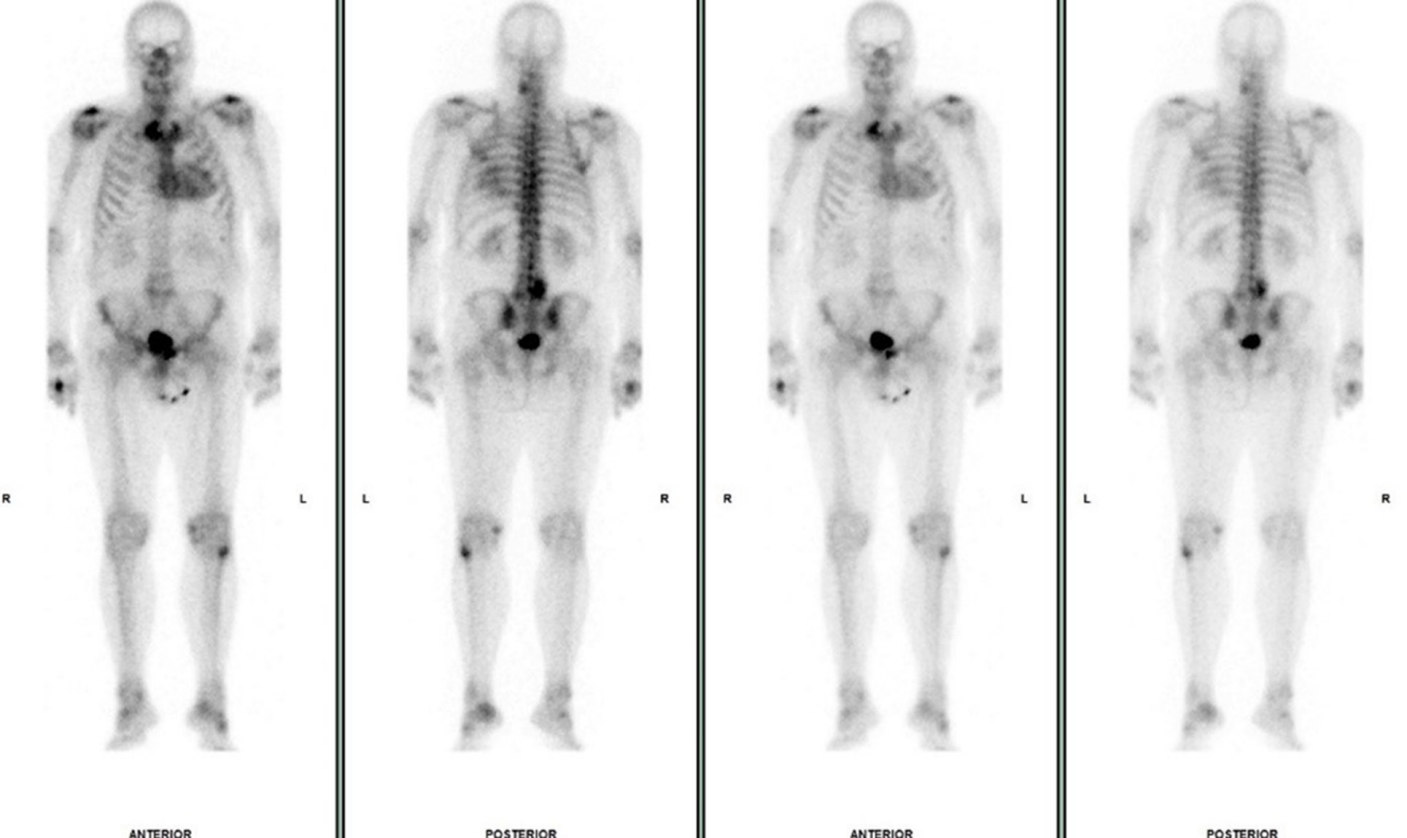
Whole body bone scan in case 2. There is incidental significant cardiac uptake raising concern for cardiac ATTR amyloidosis. Otherwise, there is expected radiotracer accumulation in the skeleton and urinary tract and additional sites of focal uptake related to degenerative disease at multiple joints. Focal uptake at the injection site in the right hand is also demonstrated.

**Figure 5.**
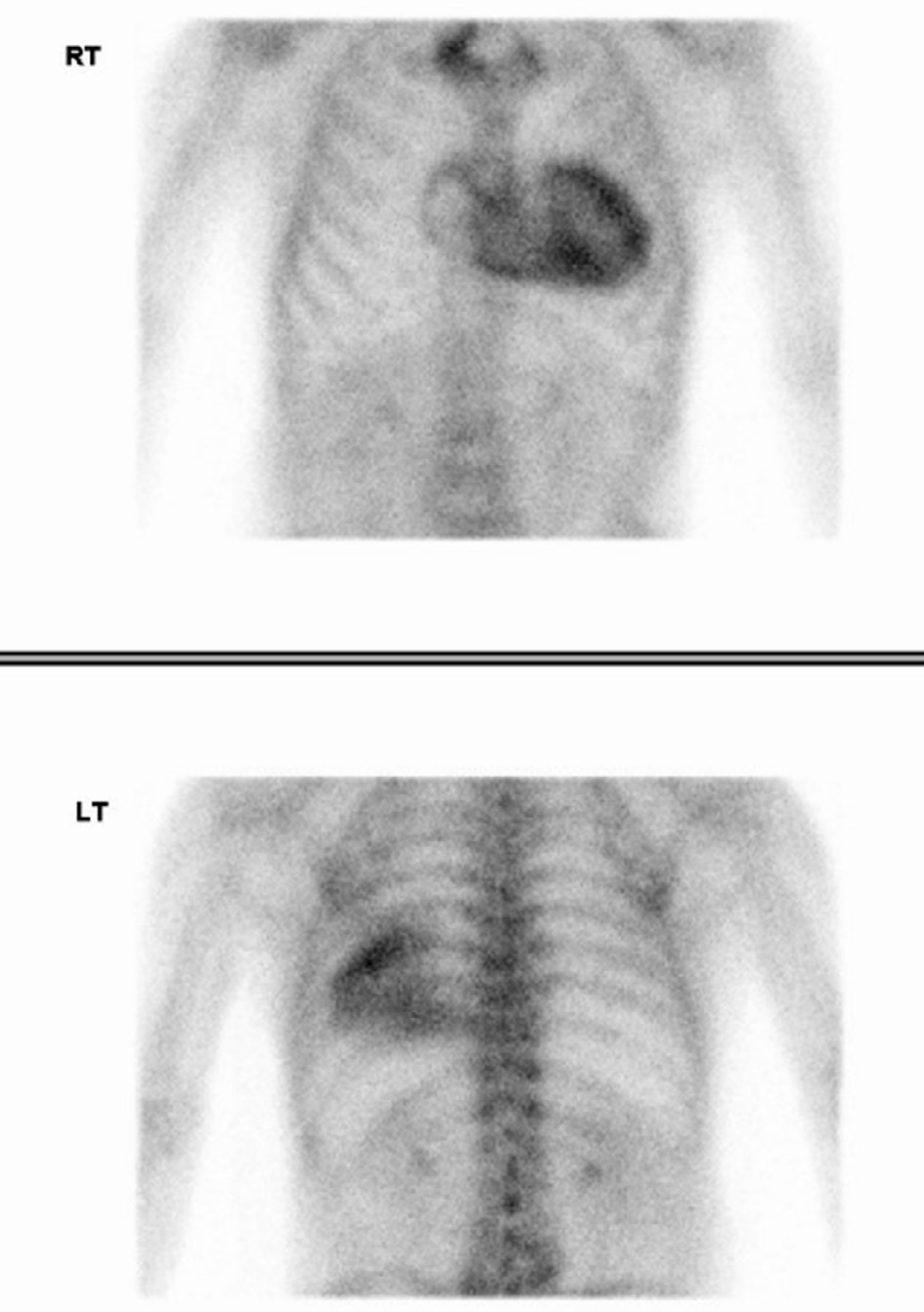
Cardiac scintigraphy in case 2. Anterior and posterior planar thoracic images, acquired 3 h after 99mTc-DPD administration, demonstrate severe myocardial uptake (Perugini 3 – uptake greater than rib uptake) predominantly in the left ventricle, compatible with the diagnosis of cardiac ATTR amyloidosis. Diagnosis was confirmed on endomyocardial biopsy in this case.

Case 2 is a 64-year-old male who underwent whole body bone scintigraphy for further investigation of a sclerotic vertebral lesion identified on CT (Figure 3). No concerning focal uptake related to the vertebral lesion was demonstrated. Significant diffuse cardiac uptake was identified, however, and was reported with the potential association with cardiac amyloidosis suggested (Figure 4). There was no known underlying cardiac disorder at this time as per retrospective review of hospital medical notes. As in case 1, subsequent clinical assessment revealed symptoms of heart failure (NHYA Class 3) and further investigation with echocardiography, serum/urine analysis and dedicated cardiac scintigraphy was performed. Echocardiography demonstrated moderate/severe left ventricular hypertrophy, restrictive diastolic dysfunction and a speckled appearance of the myocardium. These findings are suggestive of cardiac amyloidosis. On cardiac scintigraphy, there was severe myocardial DPD uptake (Perugini 3) and cardiac ATTR amyloidosis was diagnosed (Figure 5). The diagnosis was confirmed on endomyocardial biopsy in this case.

**Figure 6.**
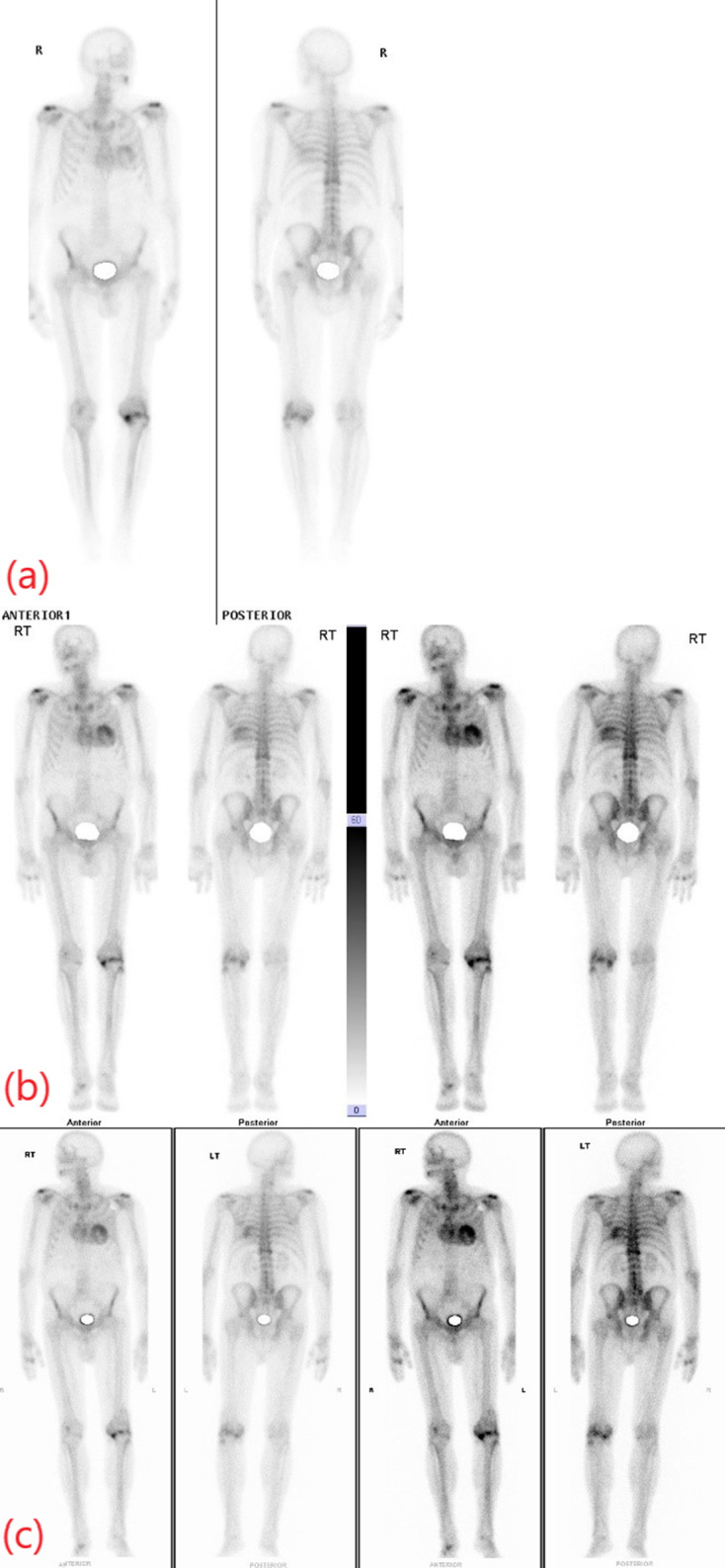
Serial whole-body bone scans in case 3. The initial scan (**a**), performed 8 years prior to cardiac ATTR amyloidosis diagnosis as part of prostate cancer staging, demonstrates mild/moderate cardiac uptake (Perugini 2). Subsequent bone scintigraphy performed 4 years (**b**) and 3 years (**c**) prior to cardiac ATTR amyloidosis diagnosis shows severe cardiac uptake (Perugini 3). A significant increase in the extent of DPD uptake is seen when compared to the initial bone scan.

**Figure 7.**
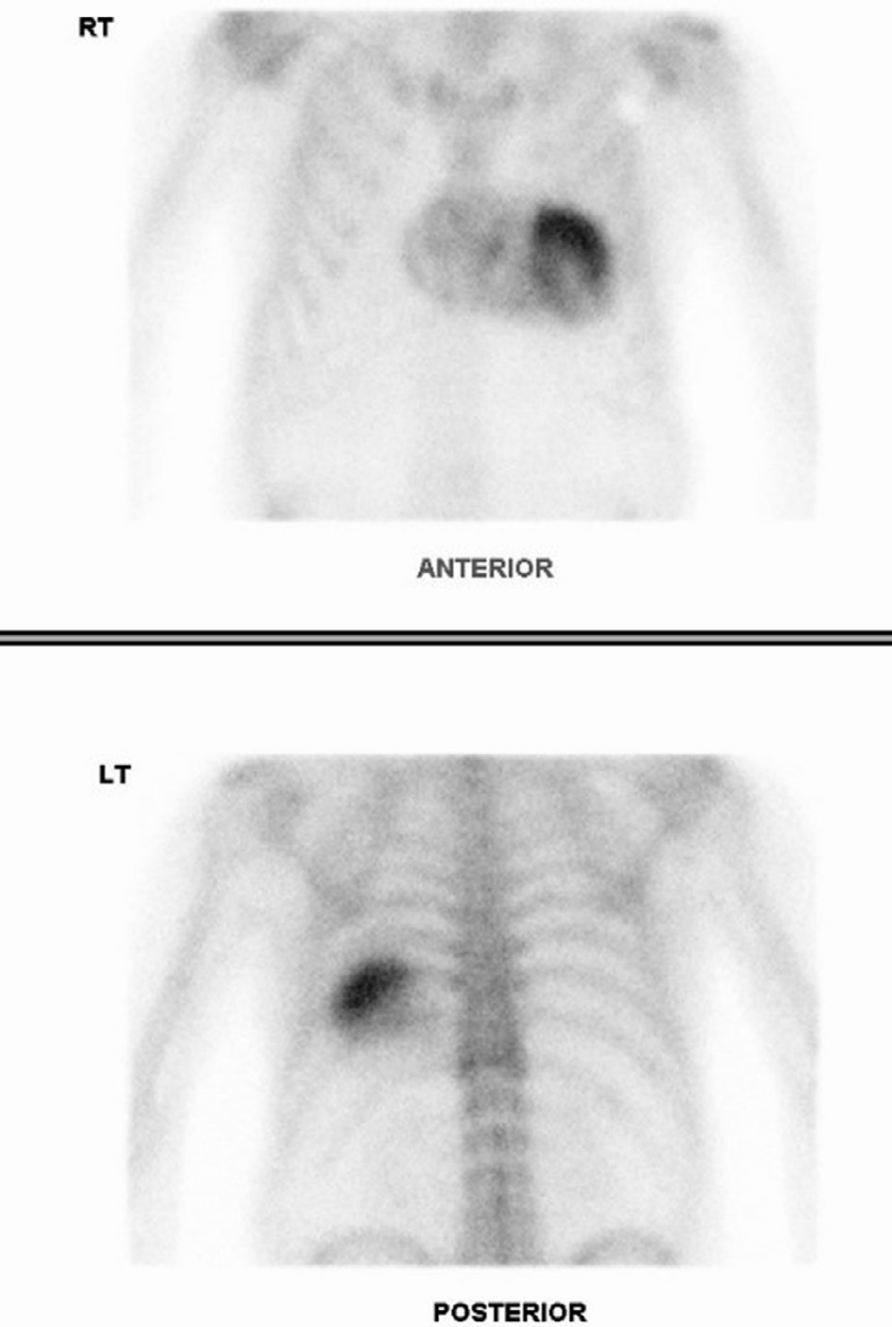
Cardiac scintigraphy in case 3. Anterior and posterior planar thoracic images, acquired 3 h after 99mTc-DPD administration, demonstrate severe myocardial uptake (Perugini 3 – uptake greater than rib uptake) predominantly in the left ventricle, compatible with the diagnosis of cardiac ATTR amyloidosis. This study was performed 8 years after initial whole-body bone scan and 3/4 years after

Case 3 is a 77-year-old male who initially underwent whole body bone scintigraphy in 2012 as part of prostate cancer staging. Mild cardiac uptake was demonstrated on the bone scan and a differential diagnosis including ischaemia/infarction and myocarditis was reported (Figure 6a). A repeat bone scan was performed in 2016 also for prostate cancer staging (Figure 6b). Relatively unchanged cardiac uptake was reported and potential association with myocardial infarction suggested. A further bone scan as part of prostate cancer restaging was done in 2017 with similar cardiac uptake described and potential association with myocardial infarction reported (Figure 6c). The patient subsequently presented with heart failure symptoms (NYHA Class 3) in 2020 and underwent echocardiography. This showed diastolic dysfunction, global longitudinal strain with apical sparing and a speckled appearance of the myocardium suggestive of cardiac amyloidosis. Testing for AL amyloidosis monoclonal gammopathy was negative. Cardiac scintigraphy demonstrated severe myocardial DPD uptake (Perugini 3) and the diagnosis of cardiac ATTR amyloidosis was made (Figure 7). TTR gene therapy was commenced, however, this was not tolerated by the patient requiring it to be discontinued.

## Discussion

Cardiac ATTR amyloidosis is an under-recognized cause of diastolic heart failure with preserved ejection fraction in elderly patients.^12,15^ While AL amyloidosis is relatively rare, ATTR amyloidosis has been identified in up to 10–16% of elderly heart failure patients and is also common in patients with severe aortic stenosis.^12,15–17^ Older autopsy studies have shown cardiac ATTR amyloid deposits in up to 25% of people over 80 years.^18,19^ The diagnosis of cardiac amyloidosis is often delayed as clinical features are varied and overlap with other causes of heart failure, findings on echocardiography and electrocardiography are non-specific and awareness of the disorder is often lacking.^14^ Endomyocardial biopsy was previously required for definitive diagnosis and while this remains the gold standard test, the ability to non-invasively diagnose ATTR amyloidosis using a combination of imaging and serum/urine analysis has been demonstrated in recent years.

The association between myocardial bone avid radiotracer uptake and cardiac amyloidosis has been recognised for nearly 40 years.^20,21^ However, it has assumed far greater importance with the discovery that it could provide a non-invasive means to differentiate between AL and ATTR amyloidosis, initially described by Perugini et al in 2005.^13,14^ Diphosphonate radiotracers used in bone scintigraphy – such as DPD, PYP and HDMP – reliably show myocardial accumulation in ATTR amyloidosis, whereas minimal or no myocardial uptake is seen in AL amyloidosis.^13,14^ Interestingly, MDP does not demonstrate the same sensitivity as other diphosphonate agents, for unknown reasons.^22^ This non-invasive distinction between AL and ATTR cardiac amyloidosis cannot be reliably achieved based on clinical, echocardiography or cardiac magnetic resonance findings.

As a result, dedicated cardiac scintigraphy has emerged a key investigation in the diagnosis of ATTR amyloidosis. As in whole-body bone scintigraphy, 99mTc labelled diphosphonate radiotracers are used and a similar dose of 370–740 MBq (10–20 mCi) is recommended.^12^ Unlike whole-body bone scans, a single image acquisition Phase 1–3 h after administration is used and images are acquired of the thorax only in anterior and posterior projections. Additionally, while SPECT is commonly not performed as part of whole bone scintigraphy, it is recommended in all cases where planar imaging is positive for cardiac uptake.^12^ SPECT helps to distinguish myocardial uptake from uptake in soft tissue or bone in the same two-dimensional plane and increases the accuracy of quantification of myocardial uptake using heart-contralateral lung ratios or other methods, which may be important for follow-up.^12^ In addition, it has been demonstrated that SPECT may identify low-grade (Grade 1) myocardial uptake, which is not visible on planar imaging in some cases of ATTR amyloidosis.^23^ Cardiac scintigraphy, therefore, has the advantages of a smaller field of view, which improves image quality and of the routine addition of SPECT in comparison with whole body scanning for assessment of myocardial uptake. With increased awareness of the potential importance of incidental cardiac uptake, a dedicated cardiac view and SPECT may be performed immediately after the conventional bone scan if myocardial uptake is observed to avoid the need for a repeat scintigraphy study. Grade 2 or 3 myocardial uptake (uptake equal to/greater than bone uptake) on cardiac scintigraphy is highly suggestive of ATTR amyloidosis and can diagnose cardiac ATTR amyloidosis with a specificity and positive predictive value of 100% where monoclonal gammopathy of AL amyloid has been excluded.^13,24^ Accordingly, cardiac scintigraphy plays a central role in the recent consensus algorithm for the non-invasive diagnosis of ATTR amyloidosis.^12,13^ It is based on this new non-invasive diagnostic approach that the high prevalence of ATTR amyloidosis in clinical settings such as heart failure with preserved ejection fraction has been shown.^12^ Furthermore, cardiac scintigraphy may identify amyloid deposition before abnormalities manifest clinically or on echocardiography or MRI facilitating earlier diagnosis.^25,26^

In addition to the assessment of cardiac uptake in comparison with the ribs, analysis of cardiac uptake relative to other bone and surrounding skeletal muscle uptake has also been described.^23^ In patients with Grade 2 or 3 cardiac uptake in ATTR amyloidosis, loss of the normal peak on the line count profile associated with uptake in the femur when a region of interest is drawn across the thigh on anterior planar imaging has been shown due to the increase in surrounding skeletal muscle uptake.^23^ Thus, the apparent decrease in bone uptake in the setting of significant myocardial accumulation may be related primarily to increasing generalised soft tissue uptake.

In the past, ATTR amyloidosis could only be successfully treated by cardiac transplantation as conventional heart failure medications are ineffective.^12^ Novel gene therapies that stabilise or suppress TTR expression have been developed, however, offering potential treatments for this morbid and previously fatal disease.^27,28^ As new treatments emerge, accurate and timely diagnosis is imperative to facilitate initiation of therapy at the early stages of the disease before severe functional impairment occurs. Indeed, cardiac scintigraphy has been suggested as a potential screening tool for identification of subclinical ATTR amyloidosis in this regard.^29^

The importance of cardiac scintigraphy in the diagnosis of ATTR amyloidosis has led to significant interest in re-evaluating incidental cardiac uptake on bone scintigraphy and multiple retrospective studies of bone scans performed for rheumatological or oncological indications in patients without known or suspected cardiac disease have been performed.^29–31^ These aim to establish the prevalence of ATTR amyloidosis as indicated by significant myocardial radiotracer accumulation, to evaluate the potential for early identification of the disease and assess the association with heart failure. A review of 12,400 bone scans, reported by Longhi et al, identified incidental myocardial uptake in 45 cases (0.36%) with prevalence increasing with age.^29^ Another study, described by Mohamed-Salem et al, which included only patients over 75 years of age showed incidental myocardial uptake in 31 of 1114 patients (2.78%) with prevalence also increasing with age.^30^ Mohamed-Salem et al also demonstrated an association between myocardial uptake and an increased risk of hospitalisation due to heart failure, supporting the link between ATTR amyloidosis and heart failure in elderly patients. Kim et al, in a retrospective analysis of bone scans with 99mTc-DPD, reported incidental cardiac uptake only 6 of 9581 cases (0.06%), although all were over 70 years giving a prevalence in this older cohort of 0.4%.^31^ These studies highlight that incidental myocardial uptake during bone scintigraphy is not that uncommon, particularly with increasing age.

Our case series demonstrates the potential to identify subclinical ATTR amyloidosis based on incidental cardiac uptake during bone scintigraphy. In case 1 and case 2, the accurate identification of significant myocardial radiotracer accumulation led to the diagnosis of cardiac ATTR amyloidosis following further investigation. In both cases, symptoms of heart failure were present but were not yet of sufficient severity to prompt specialist evaluation. Early identification of ATTR amyloidosis in these cases allowed TTR gene therapy to be initiated at an early clinical stage of disease. In contrast, incidental cardiac uptake during serial bone scintigraphy studies for prostate cancer staging was identified in case 3 but did not lead to further investigation for cardiac amyloidosis. The patient later presented, 8 years after the initial bone scan, with significant heart failure symptoms and failed to tolerate the TTR gene therapy. The diagnosis may potentially have been made at an earlier stage based on the incidental bone scan findings, highlighting the need for careful correlation of incidental myocardial uptake with the clinical history and consideration of further investigation if an obvious cause such as recent myocardial infarction or unstable angina are not present. Accurate reporting of the pattern of incidental myocardial uptake may also be of benefit as focal uptake is suggestive of ischaemia/infarction, whereas more diffuse uptake should prompt investigation for other causes.^9^

## Conclusion

Cardiac scintigraphy using bone avid radiotracers has emerged as a key investigation in the diagnosis of ATTR cardiac amyloidosis and may also help in the early detection of the disease. Novel TTR gene therapies now offer potential disease modifying treatment for ATTR amyloidosis if initiated early in the disease process before severe functional impairment occurs. Incidental cardiac uptake in whole-body bone scintigraphy must, therefore, be carefully assessed and accurately reported as there is the potential to diagnose subclinical ATTR amyloidosis and facilitate early treatment. Recent studies have shown that incidental cardiac uptake on bone scintigraphy in elderly patients is not overly uncommon and an awareness of this potential relationship with ATTR cardiac amyloidosis is important for all radiologists who may be involved in the reporting of whole-body bone scans either directly or indirectly, such as during oncological multidisciplinary meeting preparation.

## Learning points

- Incidental cardiac uptake in bone scintigraphy has taken on increased importance as a result of recent advances in the diagnosis and management of cardiac amyloidosis.
- Dedicated cardiac scintigraphy using bone avid radiotracers now plays a key role in the non-invasive diagnosis of cardiac transthyretin (ATTR) amyloidosis.
- Cardiac ATTR amyloidosis is a previously under-recognised cause of heart failure with preserved ejection fraction in elderly patients.
- Novel targeted gene therapies for cardiac ATTR amyloidosis have emerged in recent years offering promising treatment options for a previously highly morbid disease and placing great emphasis its early identification
- Incidental cardiac uptake during bone scintigraphy must be carefully assessed in conjunction with the clinical history as there is the potential to detect ATTR myocardial infiltration before the clinical manifestations of cardiac amyloidosis develop.

## References

[1] Brenner AI, Koshy J, Morey J, Lin C, DiPoce J. The bone scan. Semin Nucl Med 2012; 42: 11–26. doi: 10.1053/j.semnuclmed.2011.07.005 22117809

[2] Van den Wyngaert T, Strobel K, Kampen WU, Kuwert T, van der Bruggen W, Mohan HK, et al. The EANM practice guidelines for bone scintigraphy. Eur J Nucl Med Mol Imaging 2016; 43: 1723–38. doi: 10.1007/s00259-016-3415-4 27262701 PMC4932135

[3] Wale DJ, Wong KK, Savas H, Kandathil A, Piert M, Brown RKJ. Extraosseous findings on bone scintigraphy using fusion SPECT/CT and correlative imaging. AJR Am J Roentgenol 2015; 205: 160–72. doi: 10.2214/AJR.14.13914 26102395

[4] Bermo M, Behnia S, Fair J, Miyaoka RS, Elojeimy S. Review of extraskeletal activity on Tc-99m methylene diphosphonate bone scintigraphy and value of cross-sectional and SPECT-CT imaging correlation. Curr Probl Diagn Radiol 2018; 47: 324–32. doi: 10.1067/j.cpradiol.2017.07.009 28844319

[5] Zuckier LS, Freeman LM, Nonosseous FLM. Nonosseous, nonurologic uptake on bone scintigraphy: atlas and analysis. Semin Nucl Med 2010; 40: 242–56. doi: 10.1053/j.semnuclmed.2010.02.003 20513447

[6] Peller PJ, Ho VB, Kransdorf MJ. Extraosseous Tc-99m MDP uptake: a pathophysiologic approach. Radiographics 1993; 13: 715–34. doi: 10.1148/radiographics.13.4.8356264 8356264

[7] Caobelli F, Paghera B, Pizzocaro C, Guerra UP. Extraosseous myocardial uptake incidentally detected during bone scan: report of three cases and a systematic literature review of extraosseous uptake. Nucl Med Rev Cent East Eur 2013; 16: 82–7. doi: 10.5603/NMR.2013.0040 24068638

[8] Kaye J, Hayward M. Soft tissue uptake on 99mTc methylene diphosphonate bone scan imaging: pictorial review. Australas Radiol 2002; 46: 13–21. doi: 10.1046/j.1440-1673.2001.00989.x 11966582

[9] Codini MA, Turner DA, Battle WE, Hassan P, Ali A, Messer JV. Value and limitations of technetium-99m stannous pyrophosphate in the detection of acute myocardial infarction. Am Heart J 1979; 98: 752–62. doi: 10.1016/0002-8703(79)90474-5 495427

[10] Al-Nahhas AM, Jinnouchi S, Anagnostopoulos C, Hirsch W, Heary T, McCready VR. Clinical significance of technetium-99m methylene diphosphonate myocardial uptake: association with carcinoma of the prostate. Eur J Nucl Med 1995; 22: 148–53. doi: 10.1007/BF00838945 7758502

[11] Jones A, Keeling D. Benign myocardial uptake of hydroxymethylene diphosphonate. Nucl Med Commun 1994; 15: 21–3. doi: 10.1097/00006231-199401000-00004 8152688

[12] Dorbala S, Ando Y, Bokhari S, Dispenzieri A, Falk RH, Ferrari VA, et al. ASNC/AHA/ASE/EANM/HFSA/ISA/SCMR/SNMMI expert consensus recommendations for multimodality imaging in cardiac amyloidosis: Part 1 of 2-evidence base and standardized methods of imaging. J Nucl Cardiol 2019; 26: 2065–123. doi: 10.1007/s12350-019-01760-6 31468376

[13] Gillmore JD, Maurer MS, Falk RH, Merlini G, Damy T, Dispenzieri A, et al. Nonbiopsy diagnosis of cardiac transthyretin amyloidosis. Circulation 2016; 133: 2404–12. doi: 10.1161/CIRCULATIONAHA.116.021612 27143678

[14] Perugini E, Guidalotti PL, Salvi F, Cooke RMT, Pettinato C, Riva L, et al. Noninvasive etiologic diagnosis of cardiac amyloidosis using 99mTc-3,3-diphosphono-1,2-propanodicarboxylic acid scintigraphy. J Am Coll Cardiol 2005; 46: 1076–84. doi: 10.1016/j.jacc.2005.05.073 16168294

[15] González-López E, Gallego-Delgado M, Guzzo-Merello G, de Haro-Del Moral FJ, Cobo-Marcos M, Robles C, et al. Wild-type transthyretin amyloidosis as a cause of heart failure with preserved ejection fraction. Eur Heart J 2015; 36: 2585–94. doi: 10.1093/eurheartj/ehv338 26224076

[16] Treibel TA, Fontana M, Gilberson JA. Occult transthyretin cardiac amyloid in severe calcified aortic stenosis: prevalence and prognosis in patients undergoing surgical aortic valve replacement. Circ Cardiovasc Imaging 2016; 9: 1–11. 10.1161/CIRCIMAGING.116.005066 27511979

[17] Castaño A, Narotsky DL, Hamid N, Khalique OK, Morgenstern R, DeLuca A, et al. Unveiling transthyretin cardiac amyloidosis and its predictors among elderly patients with severe aortic stenosis undergoing transcatheter aortic valve replacement. Eur Heart J 2017; 38: 2879–87. doi: 10.1093/eurheartj/ehx350 29019612 PMC5837725

[18] Cornwell GG, Murdoch WL, Kyle RA, Westermark P, Pitkänen P. Frequency and distribution of senile cardiovascular amyloid. A clinicopathologic correlation. Am J Med 1983; 75: 618–23. doi: 10.1016/0002-9343(83)90443-6 6624768

[19] Tanskanen M, Peuralinna T, Polvikoski T, Notkola I-L, Sulkava R, Hardy J, et al. Senile systemic amyloidosis affects 25% of the very aged and associates with genetic variation in alpha2-macroglobulin and tau: a population-based autopsy study. Ann Med 2008; 40: 232–9. doi: 10.1080/07853890701842988 18382889

[20] Wizenberg TA, Muz J, Sohn YH, Samlowski W, Weissler AM. Value of positive myocardial technetium-99m-pyrophosphate scintigraphy in the noninvasive diagnosis of cardiac amyloidosis. Am Heart J 1982; 103(4 Pt 1): 468–73. doi: 10.1016/0002-8703(82)90331-3 6278906

[21] Eriksson P, Backman C, Bjerle P, Eriksson A, Holm S, Olofsson BO. Non-invasive assessment of the presence and severity of cardiac amyloidosis. A study in familial amyloidosis with polyneuropathy by cross sectional echocardiography and technetium-99m pyrophosphate scintigraphy. Br Heart J 1984; 52: 321–6. doi: 10.1136/hrt.52.3.321 6087862 PMC481632

[22] Yang JC, Fox J, Chen C, Yu AF. Cardiac ATTR amyloid nuclear imaging-not all bone scintigraphy radionuclide tracers are created equal. J Nucl Cardiol 2018; 25: 1879–84. doi: 10.1007/s12350-017-1141-3 29188431 PMC5975116

[23] Hutt DF, Quigley A-M, Page J, Hall ML, Burniston M, Gopaul D, et al. Utility and limitations of 3,3-diphosphono-1,2-propanodicarboxylic acid scintigraphy in systemic amyloidosis. Eur Heart J Cardiovasc Imaging 2014; 15: 1289–98. doi: 10.1093/ehjci/jeu107 24939945

[24] Cappelli F, Gallini C, Di Mario C, Costanzo EN, Vaggelli L, Tutino F, et al. Accuracy of 99mTc-Hydroxymethylene diphosphonate scintigraphy for diagnosis of transthyretin cardiac amyloidosis. J Nucl Cardiol 2019; 26: 497–504. doi: 10.1007/s12350-017-0922-z 28537040

[25] Rapezzi C, Quarta CC, Guidalotti PL, Pettinato C, Fanti S, Leone O, et al. Role of 99mTc-DPD scintigraphy in diagnosis and prognosis of hereditary transthyretin-related cardiac amyloidosis. JACC Cardiovasc Imaging 2011; 4: 659–70. doi: 10.1016/j.jcmg.2011.03.016 21679902

[26] Glaudemans AWJM, van Rheenen RWJ, van den Berg MP, Noordzij W, Koole M, Blokzijl H, et al. Bone scintigraphy with (99m)technetium-hydroxymethylene diphosphonate allows early diagnosis of cardiac involvement in patients with transthyretin-derived systemic amyloidosis. Amyloid 2014; 21: 35–44. doi: 10.3109/13506129.2013.871250 24455993

[27] Maurer MS, Schwartz JH, Gundapaneni B, Elliott PM, Merlini G, Waddington-Cruz M, et al. Tafamidis treatment for patients with transthyretin amyloid cardiomyopathy. N Engl J Med 2018; 379: 1007–16. doi: 10.1056/NEJMoa1805689 30145929

[28] Adams D, Gonzalez-Duarte A, O'Riordan WD, Yang C-C, Ueda M, Kristen AV, et al. Patisiran, an RNAi Therapeutic, for hereditary transthyretin amyloidosis. N Engl J Med 2018; 379: 11–21. doi: 10.1056/NEJMoa1716153 29972753

[29] Longhi S, Guidalotti PL, Quarta CC, Gagliardi C, Milandri A, Lorenzini M, et al. Identification of TTR-related subclinical amyloidosis with 99mTc-DPD scintigraphy. JACC Cardiovasc Imaging 2014; 7: 531–2. doi: 10.1016/j.jcmg.2014.03.004 24831216

[30] Mohamed-Salem L, Santos-Mateo JJ, Sanchez-Serna J, Hernández-Vicente Álvaro, Reyes-Marle R, Castellón Sánchez MI, et al. Prevalence of wild type ATTR assessed as myocardial uptake in bone scan in the elderly population. Int J Cardiol 2018; 270: 192–6. doi: 10.1016/j.ijcard.2018.06.006 29903517

[31] Kim HM, Sohn D-W, Paeng JC. Prevalence of positive 99mTc-DPD scintigraphy as an indicator of the prevalence of wild-type transthyretin amyloidosis in the elderly. Int Heart J 2019; 60: 643–7. doi: 10.1536/ihj.18-345 31019172

